# A positive effect of Cerebrolysin on motor functions and spasticity in ALS with limb or bulbar onset is questionable

**DOI:** 10.25122/jml-2024-1004

**Published:** 2024-02

**Authors:** Alfredo José Firstenfeld

**Affiliations:** 1 Servicio de Neurociencias, Universidad de Buenos Aires, Instituto Cardiológico Banfield, Buenos Aires, Argentina

## DEAR EDITOR,

We appreciate the opportunity to address the comments and questions raised regarding our ALS study [1]. We investigated the efficacy of Cerebrolysin as add-on treatment in patients with ALS.

Regarding the short duration, we understand that it is questionable for the readers, but not for the authors, because what we saw in our study was that motor function and spasticity were modified, and both improved after treatment with Cerebrolysin.

Regarding the comment about the crossover phase, we would like to explain that the original protocol was only designed for a 3-month follow-up period. We look forward to continuing with a second phase, which will include a larger number of patients and a longer follow-up period in addition to the crossover.

In this first protocol, the inclusion criteria were patients with confirmed ALS according to the El Escorial criteria, with all clinical types (limb and bulbar onset). All patients included in the protocol were of sporadic type, no familial candidates were found.

Owing to the advanced course of the disease, all patients had motor deficits of the upper and lower limbs. Furthermore, our patients could not bend their knees due to spasticity that fixed the knee.

Most patients were given a vitamin and protein diet.

Currently, there is no treatment that modifies the signs or symptoms of ALS. The results we obtained after treating patients with ALS with an average disease duration of 18 months and significant motor function impairments (swallowing, speech, upper and lower limb movement) with Cerebrolysin are very encouraging. For this reason, we would like to emphasize the importance of further testing of this drug.

Our aim is to continue with a new long-term study that includes different clinical types and different stages of development (initial and advanced) of ALS, because our previous observations showed a rapid and positive improvement after treatment.
